# Low-Profile Proximity-Coupled Cavity-Less Magneto-Electric Dipole Antenna

**DOI:** 10.3390/s25041234

**Published:** 2025-02-18

**Authors:** Khalid Almegbel, Kin-Fai Tong

**Affiliations:** 1 Department of Electronic and Electrical Engineering, University College London, Torrington Place, London WC1E 7JE, UK; k.tong@ucl.ac.uk; 2 King Abdulaziz City for Science and Technology, Riyadh 11442, Saudi Arabia

**Keywords:** proximity coupled, suspended coupling line, wideband, stable high gain, magneto-electric dipoles

## Abstract

Magneto-electric dipole (ME-dipole) antennas offer several advantages, including wide impedance bandwidth, stable high gain, unidirectional radiation, and low back-lobe radiation patterns, making them suitable for modern wireless communication systems. However, the thickness of conventional ME-dipole antennas is typically about a quarter wavelength (0.25$\lambda_{o}$) at the center operating frequency, which may not be desirable for portable device applications. This work introduces a new feeding method that reduces the antenna profile and ground plane size while maintaining the same advantages. A suspended horizontal line is proposed to excite the cavity-less ME-dipole antenna through proximity coupling. The measured results demonstrate a wide impedance bandwidth of 45.3% (ranging from 2.05 GHz to 3.25 GHz) and an average in-band gain of 9 dBi with stable ±1 dBi in-band variation with a ground reflector of size about 0.89$\lambda_{o}^{2}$. More importantly, the cavity-less design reduces the overall thickness of the antenna to 0.17$\lambda_{o}$ at the center operating frequency.

## 1. Introduction

The conventional ME-dipole antennas reported in the literature, with different geometries and feeding methods such as L-shaped or Γ-shaped feeding structures, have an overall structure thickness of about $0.25\lambda_{0}$, as the feed lines and cavity walls are utilized to excite the magnetic dipole (M-dipole) mode [1,2,3,4]. In [1], a novel feeding method utilizing a Γ-shaped strip was introduced to simultaneously excite a pair of planar sheets forming the electric dipole and a vertically oriented quarter-wavelength shorted patch antenna serving as the magnetic dipole. This innovative design achieved a wide impedance bandwidth of 43.8%, spanning from 1.85 GHz to 2.89 GHz. The Γ-shaped strip feed line not only facilitated the wide bandwidth but also ensured low cross-polarization, symmetrical E- and H-plane radiation patterns, low back-lobe radiation, and high performance. The antenna achieved a high gain of 7.5 dBi with a stable in-band gain variation of ±0.5 dB, making it highly suitable for wideband wireless communication systems.

To enhance the performance of the wideband ME-dipole antenna discussed in [1], the authors in [2] proposed introducing a vertical slot into the vertically oriented quarter-wavelength shorted patch antenna, forming a defected ground structure (DGS). The incorporation of the DGS effectively reduces the electric field intensity, primarily decreasing the capacitance of the air-gap microstrip line. This reduction in capacitance minimizes the variation in the imaginary component of the impedance, thereby improving the stability of the feeding network’s impedance. This modification significantly extended the antenna’s impedance bandwidth from 43% to 87% and increased the maximum gain to 8.3 dBi.

Several antennas have been reported in the literature to achieve dual-band magneto-electric dipole operation. In [3], a dual-polarized ME-dipole antenna employing a Γ-shaped feeding system is introduced to excite two orthogonal modes. This antenna achieved a wide impedance bandwidth of 66%, a high gain of 9.5 dBi, and a high isolation of 36 dB, though its thickness was approximately $0.23\lambda_{0}$. In [5], a wideband dual-band magneto-electric dipole antenna with an enhanced feeding structure is presented. The design offers impedance bandwidths of 72% (1.48–3.15 GHz) and 21% (4.67–5.78 GHz). It features a U-shaped electric dipole and a folded shorted patch as the magnetic dipole, improving impedance matching and overall performance. The antenna also exhibited stable unidirectional radiation patterns, low cross-polarization, and minimal back-radiation, making it suitable for various wireless communication applications. In [6], a dual-band dipole antenna for base station applications is proposed. This design incorporates an irregular shorted patch and planar dipole elements, achieving two frequency bands: 0.78–1.1 GHz (34% bandwidth) and 1.58–2.62 GHz (49.5% bandwidth). It delivered stable gains of 7 dBi and 8 dBi in the respective bands. Notable features, such as an L-shaped feeding strip and a V-slot, contribute to effective impedance matching.

To provide an alternative feeding method and simplify the antenna structure, the author in [7] introduced a novel antenna geometry and feeding method offering significant improvement over previous designs. The novel design achieves a compact height of $0.097\lambda_{0}$ by replacing the original shorted quarter-wave patch with an obtuse-triangular structure while maintaining the equilateral-triangular loop for the magnetic dipole. This innovative approach eliminates the need for a coaxial cable or the Γ-shaped feed by utilizing a 50 Ω transmission line to feed the magneto-electric (ME) dipole. This antenna maintains the key benefits of the original ME dipole design, including wide impedance bandwidth, high gain, low cross-polarization, and low back-lobe radiation, while simplifying the overall structure. In [8,9], the authors proposed a novel magneto-electric dipole antenna design that eliminates the need for a vertical quarter-wavelength slot cavity. The antenna in [8] is low-profile and achieved outstanding radiation characteristics, including a maximum gain of 9.74 dBi with 1.8 dB gain variation within the bandwidth and an impedance bandwidth of 41.03%. Notably, the antenna exhibits similar radiation patterns in both the E-plane and H-plane, simplifying implementation and reducing production costs by utilizing two rectangular patches, a coaxial cable, and a ground reflector.

In this paper, a novel cavity-less magneto-electric (ME) dipole antenna is proposed. The design utilizes a suspended transmission line to excite the electric dipole (E-dipole) and magnetic dipole (M-dipole) modes through proximity coupling. The operating frequencies of the E-dipole and M-dipole are separated, with the E-dipole mode resonating in the lower frequency band and the M-dipole mode resonating in the higher band. This separation enables the antenna to achieve a wide impedance bandwidth and stable high realized gain. The feeding structure consists of a vertical semi-rigid coaxial cable and a horizontal flat transmission line, which excites the M-dipole mode without requiring quarter-wavelength cavity walls. Consequently, the antenna achieves a low profile of approximately $0.17\lambda_{0}$. The proposed ME dipole antenna achieves a wide impedance bandwidth of 53.3% in simulations, 45.3% in measurements and an average in-band gain of 9 dBi with stable ±1 dBi in-band variation with a ground reflector with a size of about 0.89$\lambda_{o}^{2}$. Furthermore, due to the reduced profile, the fringing field of the E-dipole mode is less extended. This innovative feeding method allows the size of the ground reflector to be reduced to $0.78\lambda_{0}$, while maintaining a wide impedance bandwidth and stable high gain.

In Section 2, an initial model with reasonable impedance bandwidth and gain is described. The model is used in Section 3 and Section 4 to explain the operating principle and optimization process. The experimental results of the optimized model are then presented and compared to other reported ME-dipole antennas in Section 5. Finally, a conclusion is provided in Section 6.

## 2. Antenna Geometry and Design

**Figure 1:**
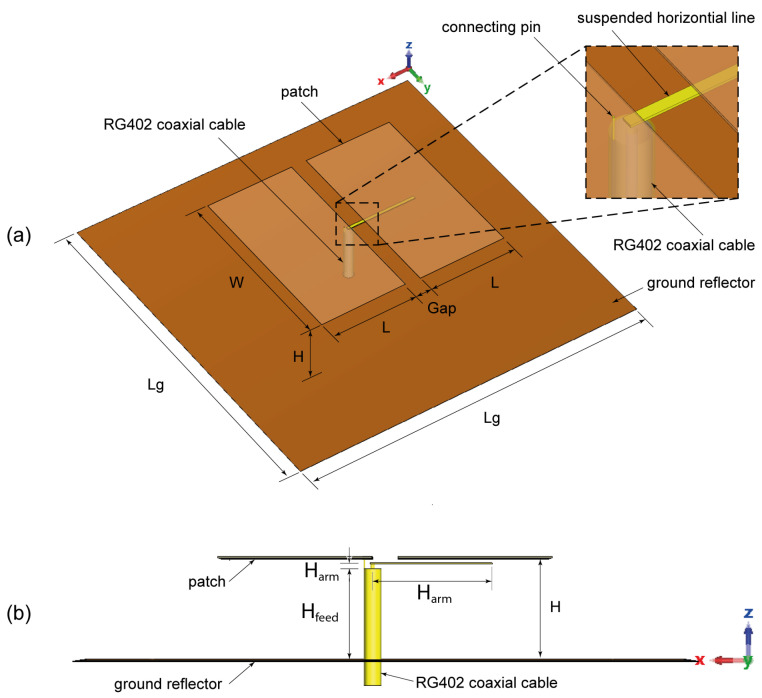
Geometry of the suspended-line coupling-fed ME-dipole antenna: (**a**) prospective view, (**b**) side view [10].

As illustrated in Figure 1, the proposed proximity-coupled cavity-less ME-dipole antenna consists of two metallic rectangular patches fed by a horizontally oriented suspended coupling line, which is extended from the inner conductor of the semi-rigid coaxial cable (RG402, 50 Ω, *⌀* 3.58 mm) and a square ground reflector. The proposed feeding method is different from the conventional L-shaped or Γ-shaped feeding system, in which the vertical part of the line of the feeding system is inductive. We propose to extend the inner conductor of the coaxial cable by a horizontally suspended line to feed the ME-dipole antenna through proximity coupling. The outer conductor of the coaxial cable is connected to the ground reflector on one end and to the edge of the patch above using a pin on the other end. The dimensions of the initial antenna parameters are listed in Table 1.

## 3. Operating Principle

**Figure 2:**
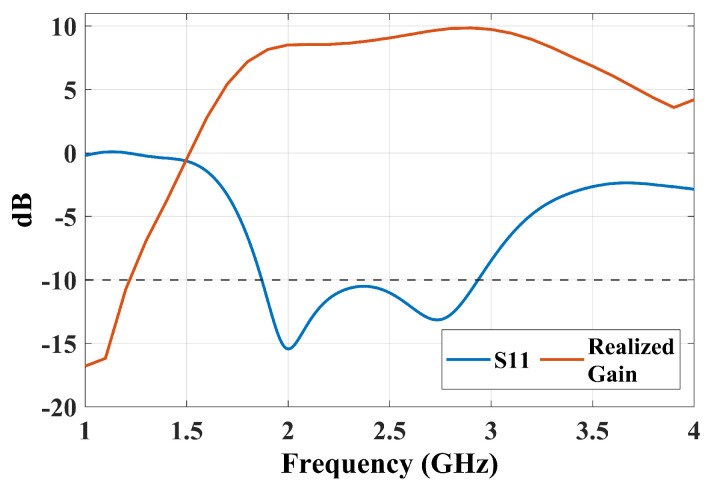
The initial results of the antenna performance $S_{11}$ and realized gain.

**Figure 3:**
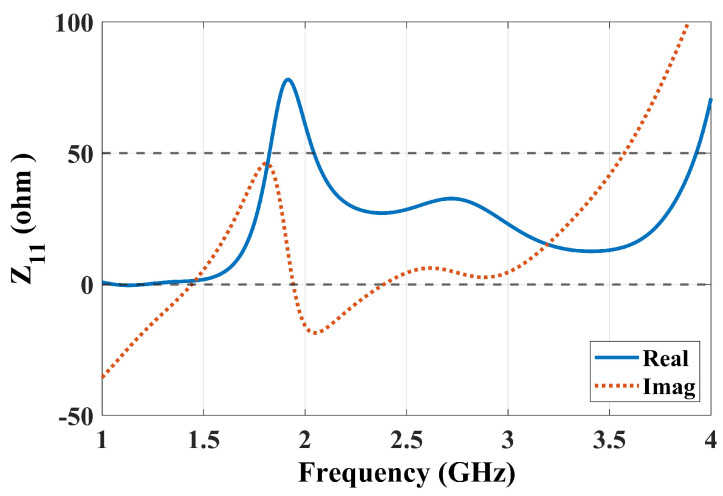
Initial results of the antenna performance $Z_{11}$.

**Figure 4:**
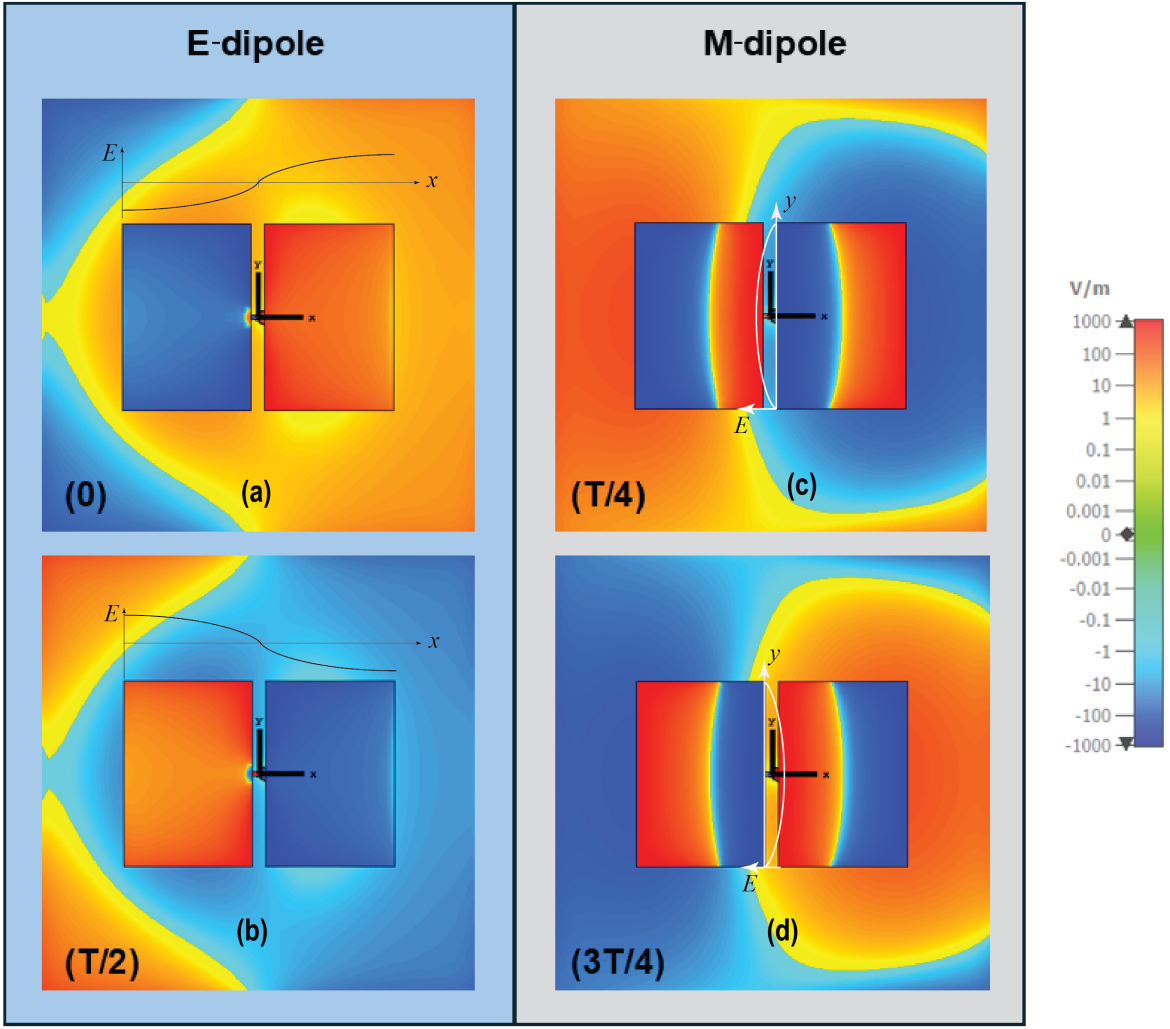
E-field distributions of the ME-dipole antenna at different time periods. E-dipole mode (**a**) t = 0, (**b**) t = T/2 and M-dipole mode (**c**) t = T/4 and (**d**) t = 3T/4.

The basic principle of ME-dipole antennas is that the E- and M-dipoles are oriented orthogonally and excited with equal power and phase [11]. This arrangement can provide wide impedance bandwidth, stable high gain, and unidirectional and symmetrical radiation patterns in both the E-plane and H-plane. In the proposed suspended-line coupling-fed ME-dipole design, the patch length (L) was set to about 0.25$\lambda_{0}$, where $\lambda_{0}$ is the wavelength at the operating frequency, for exciting the E-dipole mode, while the patch width (W) was chosen as 0.5$\lambda_{0}$, as shown in Figure 1. In this arrangement, the open-end slot located at the gap between the two arms of the E-dipole will form an M-dipole operating in a similar frequency range. The initial results demonstrate a reasonably wide impedance bandwidth and a high realized gain of about 47% and 9 dBi, respectively, as shown in Figure 2. Due to the fringing field between the two ends of the E-dipole and the ground reflector, the E-dipole exhibits a longer equivalent electrical length than the M-dipole. Figure 3 shows the antenna impedance $Z_{11}$ of the antenna, with two resonances that can be observed: one at a lower frequency around 1.9 GHz due to the E-dipole mode and another at a higher frequency of 2.7 GHz corresponding to the M-dipole mode. To further verify the principle, the electric field distribution of the antenna at 2.5 GHz is illustrated in Figure 4. The reason for choosing 2.5 GHz is that it is in the middle of the resonant frequency of the two modes. From Figure 4, we can clearly see that the E-dipole mode at t = 0 or T/2 (T is the period of the oscillating signal) and t = T/4 or 3T/4, the M-dipole mode, dominate the field distribution. The proposed ME dipole antenna was designed and simulated using CST STUDIO SUITE [12].

## 4. Parametric Study

In this section, a comprehensive analysis is presented to demonstrate the antenna’s performance in terms of different antenna parameters. The parameters are divided into two groups. The first group includes the patch length (L), patch width (W), and gap width (Gap), as the E-dipole mode is primarily determined by the patch length (L), while the M-dipole mode is influenced by the patch width (W) and slot gap width (Gap). The second group includes height (H), suspended transmission feed line length ($L_{arm}$), feed line height ($H_{arm}$) from the open-end of the semi-rigid cable, and the length of the square ground reflector ($L_{g}$). In the suspended horizontal transmission line, which is responsible for adjusting and activating the ME-dipole modes using the proposed feeding method, these parameters are important for optimizing the simultaneous excitation of both E- and M-dipole modes. In the proposed proximity coupling method, the feed line does not directly connect to the radiating elements. Instead, energy is transferred through proximity coupling between the suspended transmission feed line and the antenna patches, rather than through direct connection. This coupling method induces currents in the radiating elements, which simultaneously excites both the E- and M-dipoles. The efficiency of this coupling is highly dependent on the spacing and positioning of the feed line relative to the antenna elements. When optimized, proximity coupling can provide a much wider impedance bandwidth compared to direct feeding methods.

We begin by investigating the patch length (L) of the ME-dipole patches responsible for exciting the E-dipole mode. Next, we will investigate the patch width (W) of the ME-dipole patches and the gap between the patches (Gap) that drive the M-dipole before exploring the importance of the separation (H) between the ground plane and radiating patches. Furthermore, we investigate the impact of ground reflector dimensions ($L_{g}$) on the ME-dipole and then shift our focus to study the length of the horizontal feed line ($L_{arm}$). Finally, the spacing ($H_{arm}$) between the antenna patches and the feed line will be examined. Through this systematic approach, we aim to gain a comprehensive understanding of how these parameters collectively influence and optimize the antenna’s performance. It is noteworthy that all other antenna parameters were kept consistent with those outlined in Table 1 while studying the specified antenna parameter.

### 4.1. Patch Length (L)

**Figure 5:**
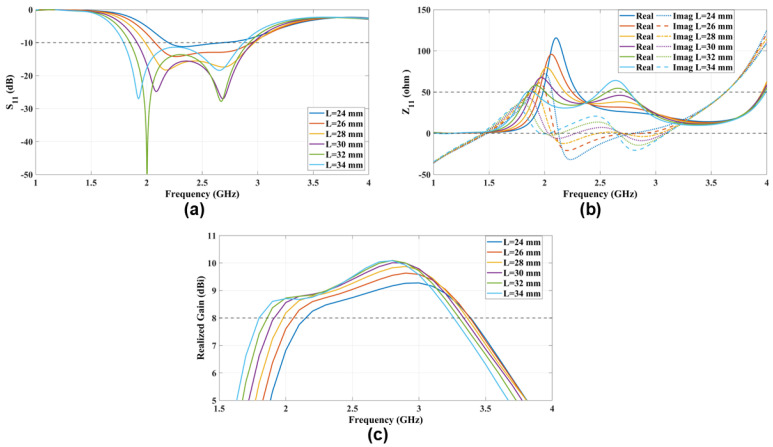
The radiation characteristics (**a**) $S_{11}$ and (**b**) $Z_{11}$ and (**c**) the realized gain parametric study that explores the impact of varying patch lengths (L) on antenna performance.

The electrical dipole characteristics are determined by the patch length (L). Specifically, at the center frequency of 2.5 GHz, the length of the E-dipole arm is expected to be 30 mm (0.25$\lambda_{0}$). The ME-dipole modes will be excited at a specific length (L), as shown in Figure 5. Since the patch length (L) is considerably shorter than the slot width (W), the M-dipole is not excited until 2L≥W. Additionally, it is observed that as the patch length (L) increases, the resonance of the E-dipole is gradually shifted to a lower frequency. The comprehensive analysis is further supported by the antenna impedance ($Z_{11}$) and the realized gain in the same figure confirming the initial analysis of the patch length (L).

### 4.2. Patch Width (W)

**Figure 6:**
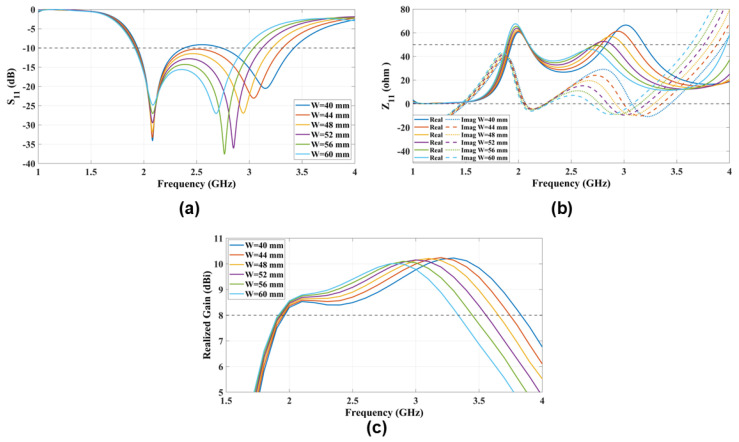
The radiation characteristics (**a**) $S_{11}$ and (**b**) $Z_{11}$ and (**c**) the realized gain parametric study that explores the impact of varying patch widths (W) on antenna performance.

The M-dipole characteristics are determined by the width of the patches (W) and the gap width (Gap). In the previous section, we discussed the role of the patch length (L) in adjusting the lower frequency resonance of the ME-dipole. In this section, we focus on the antenna patch width (W). Figure 6 shows the effect of the antenna width on the higher frequency resonance of the ME-dipole. The M-dipole resonance shifts to a higher frequency band as the patch width (W) of the patches decreases, which increases the impedance bandwidth of the ME-dipole. This comprehensive analysis is further supported by the antenna impedance ($Z_{11}$) and the realized gain in the same figure confirming the initial analysis of the patch width (W).

### 4.3. Slot Gap Width (Gap)

**Figure 7:**
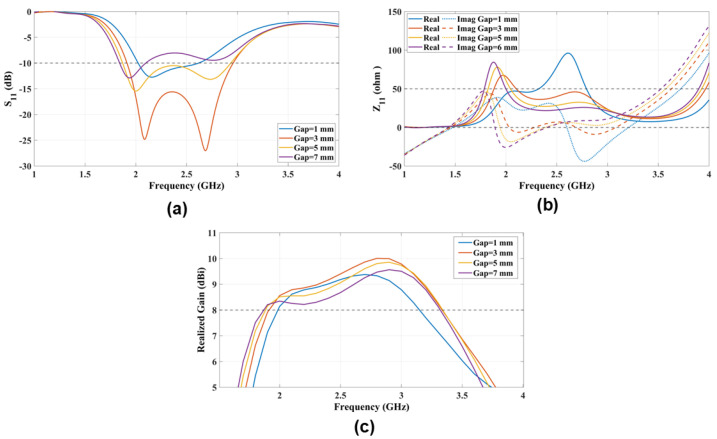
The radiation characteristics (**a**) $S_{11}$ and (**b**) $Z_{11}$ and (**c**) the realized gain parametric study that explores the impact of varying gap width (Gap) on antenna performance.

The slot width (Gap) between the patches that generate the M-dipole mode in the ME-dipole is the second key parameter influencing the activation of the M-dipole. After examining the roles of the patch width and patch length (*W* and *L*) in the previous sections, we now focus on the gap width (Gap). Figure 7 shows $S_{11}$, antenna impedance ($Z_{11}$), and the realized gain of the ME-dipole with different gap widths (Gap). The figure indicates that $\mathrm{Gap}=5\;\mathrm{mm}=0.04\lambda_{0}$ is required to generate the ME-dipole modes.

### 4.4. The Height Between the Antenna Patches and the Ground Reflector (H)

**Figure 8:**
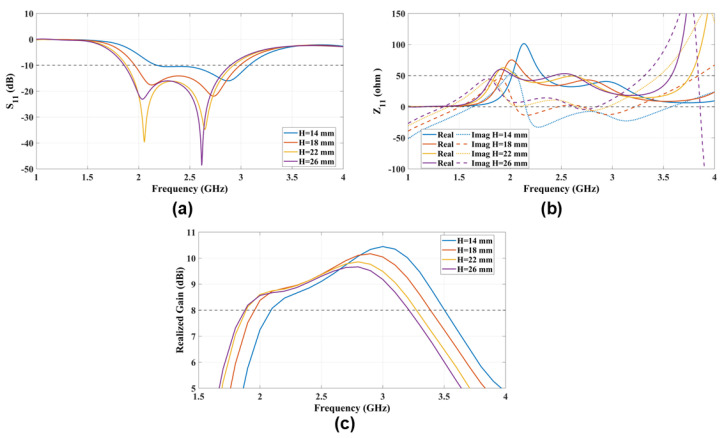
The radiation characteristics (**a**) $S_{11}$ and (**b**) $Z_{11}$ and (**c**) the realized gain parametric study that explores the impact of varying antenna height (H) on antenna performance.

The ME-dipole ground reflector is positioned at a distance of approximately $0.25\lambda_{0}$ from the radiating patches that generate the E- and M-dipoles. The ground reflector serves to make the ME-dipole unidirectional and to reduce back-lobe radiation. The spacing (*H*) between the ground reflector and the radiating patches is crucial for adjusting the ME-dipole modes, as it influences the resonant frequency of the antenna. Figure 8 shows the $Z_{11}$ of the antenna, illustrating the E- and M-dipole modes for different values of (*H*). Specifically, the E-dipole mode resonates at $H\geq 18\;\mathrm{mm}=0.15\lambda_{0}$ due to the effect of coupling with the ground reflector, as indicated by $S_{11}$. Additionally, it is important to note that the maximum realized gain will shift to a higher frequency band as the antenna height (*H*) decreases.

### 4.5. The Ground Reflector Side Dimension ($L_{g}$)

**Figure 9:**
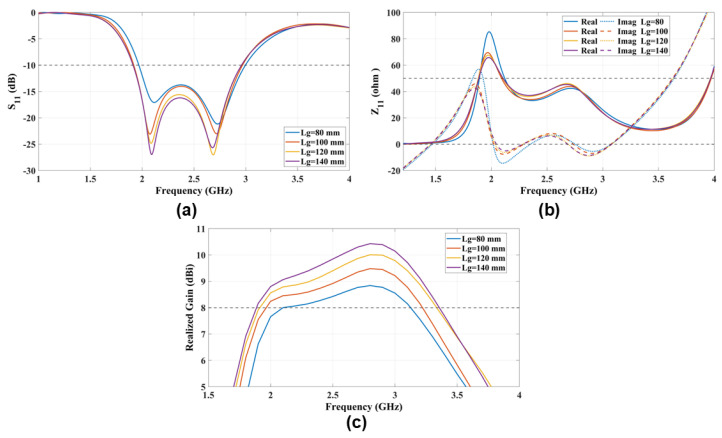
The radiation characteristics (**a**) $S_{11}$ and (**b**) $Z_{11}$ and (**c**) the realized gain parametric study that explores the impact of varying antenna ground reflector side dimension ($L_{g}$) on antenna performance.

The ground reflector is a square-shaped copper sheet with a side dimension ($L_{g}$) positioned approximately $0.25\lambda_{0}$ from the radiating patches. To achieve a balanced radiation characteristic with a unidirectional pattern, minimal back lobes, and an acceptable front-to-back ratio, it is essential to use a ground reflector with a side dimension of $L_{g}=1$ to $1.3\lambda_{0}$ for the ME-dipole antenna. Furthermore, the ground reflector is critical in the E-dipole mode due to fringing field coupling, as shown in $S_{11}$ and $Z_{11}$ in Figure 9a,b. The realized gain increases with the size of the ground reflector. The proposed ME-dipole can achieve a realized gain of 8 dBi across the operational bandwidth with a ground reflector side dimension of $L_{g}=80$ mm $=0.66\lambda_{0}$, as shown in the realized gain figure.

**Figure 10:**
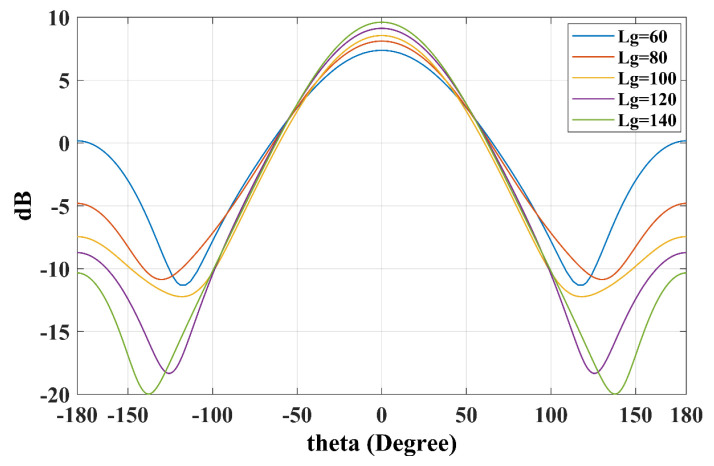
The H-plane radiation patterns with different ground sizes ($L_{g}$).

**Figure 11:**
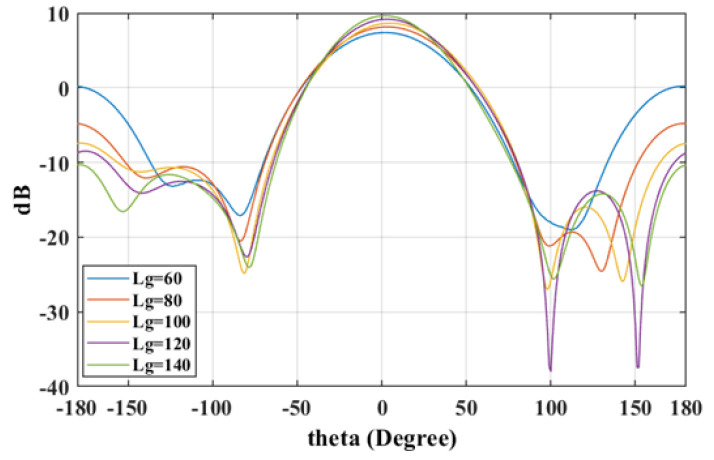
The E-plane radiation patterns with different ground sizes ($L_{g}$).

The radiation patterns in Figure 10 and Figure 11 illustrate that a reduction in ground plane size results in notably amplified back lobes, thereby reducing the front-to-back ratio, whereas larger ground planes enhance this ratio.

### 4.6. Length of the Suspended Coupling Line ($L_{arm}$)

**Figure 12:**
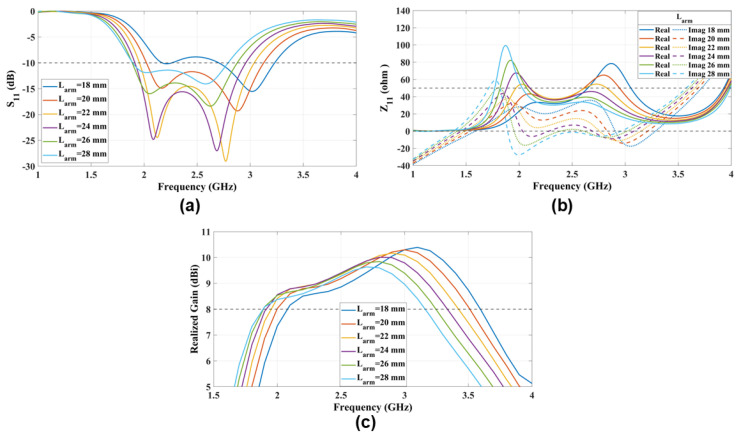
The radiation characteristics (**a**) $S_{11}$ and (**b**) $Z_{11}$ and (**c**) the realized gain parametric study that explores the impact of varying the suspended feed line length ($L_{arm}$) on antenna performance.

The proposed suspended-line coupling-fed ME-dipole is fed by a horizontally oriented suspended transmission feed line, which is extended from the inner conductor of a coaxial cable. The width of the transmission line was specifically set to 1 mm to match the coaxial cable. The antenna’s performance depends on the transmission line length ($L_{\mathrm{arm}}$), which was carefully selected to excite both the E- and M-dipole modes. To excite both modes, a transmission line length of $18\;\mathrm{mm}=0.15\lambda_{0}<L_{\mathrm{arm}}<27\;\mathrm{mm}=0.23\lambda_{0}$ was required, as shown by $S_{11}$ and the antenna impedance $Z_{11}$ in Figure 12. At $L_{\mathrm{arm}}=18\;\mathrm{mm}=0.15\lambda_{0}$, $Z_{11}$ indicates the excitation of mainly the M-dipole mode. Conversely, for $L_{\mathrm{arm}}$ values greater than or equal to $28\;\mathrm{mm}=0.23\lambda_{0}$.

### 4.7. Suspended Coupling Line Height from the Coaxial Cable ($H_{arm}$)

**Figure 13:**
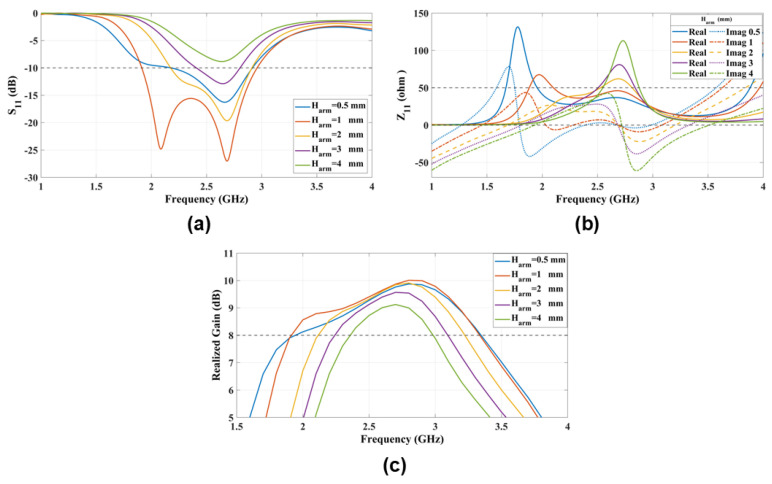
The radiation characteristics (**a**) $S_{11}$ and (**b**) $Z_{11}$ and (**c**) the realized gain parametric study that explores the impact of varying the horizontal suspended coupling line position ($H_{arm}$) on antenna performance.

The proposed feeding method uses a horizontally oriented suspended coupling line positioned at a distance $H_{\mathrm{arm}}$ below the antenna patches to excite the E- and M-dipole modes. The feed line needs to be 1 mm above the vertical coaxial cable to effectively excite the ME-dipole modes. Figure 13 shows $S_{11}$, $Z_{11}$, and the realized gain of the antenna for different $H_{\mathrm{arm}}$ heights. At $H_{\mathrm{arm}}=4$ mm, the M-dipole mode is only excited in the higher frequency band. The ME-dipole modes are excited as $H_{\mathrm{arm}}$ is reduced to 1 mm.

### 4.8. Parametric Study Summary

The parametric study investigates various key parameters affecting the performance of the proposed ME-dipole antenna. The patch length (*L*) primarily determines the E-dipole characteristics, with a patch length of 30 mm ($0.25\lambda_{0}$) resonating the E-dipole at 2.5 GHz, while the M-dipole requires 2L≥W for activation. Similarly, the width of the patches (*W*) influences the M-dipole characteristics, shifting resonance to higher frequencies with narrower patches, thereby increasing the impedance bandwidth. Moreover, the gap width (Gap) between patches is crucial for activating M-dipole modes, requiring a 5 mm ($0.04\lambda_{0}$) gap for resonance. The height (*H*) between the ground reflector and radiating patches affects the resonant frequency, with the E-dipole mode resonating at H≥18 mm ($0.15\lambda_{0}$) and realized gain shifting to higher frequencies with decreased *H*. Additionally, the size of the ground reflector ($L_{g}$) significantly impacts the radiation pattern and front-to-back ratio, achieving balance with $L_{g}$ sizes between 1 and 1.3$\lambda_{0}$, enhancing the realized gain and front-to-back ratio with larger $L_{g}$. Furthermore, the transmission line length ($L_{\mathrm{arm}}$) influences the dipole. The optimized dimensions of the ME-dipole antenna are provided in Table 2.

## 5. Results and Discussions

**Figure 14:**
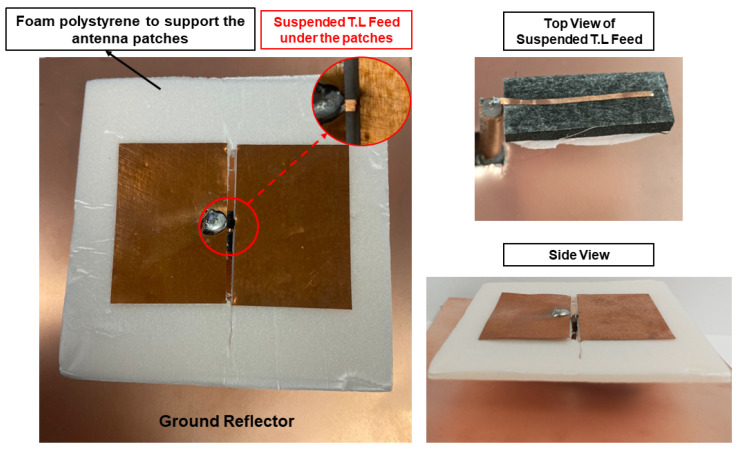
The low-profile proximity-coupled cavity-less ME-dipole antenna prototype.

**Figure 15:**
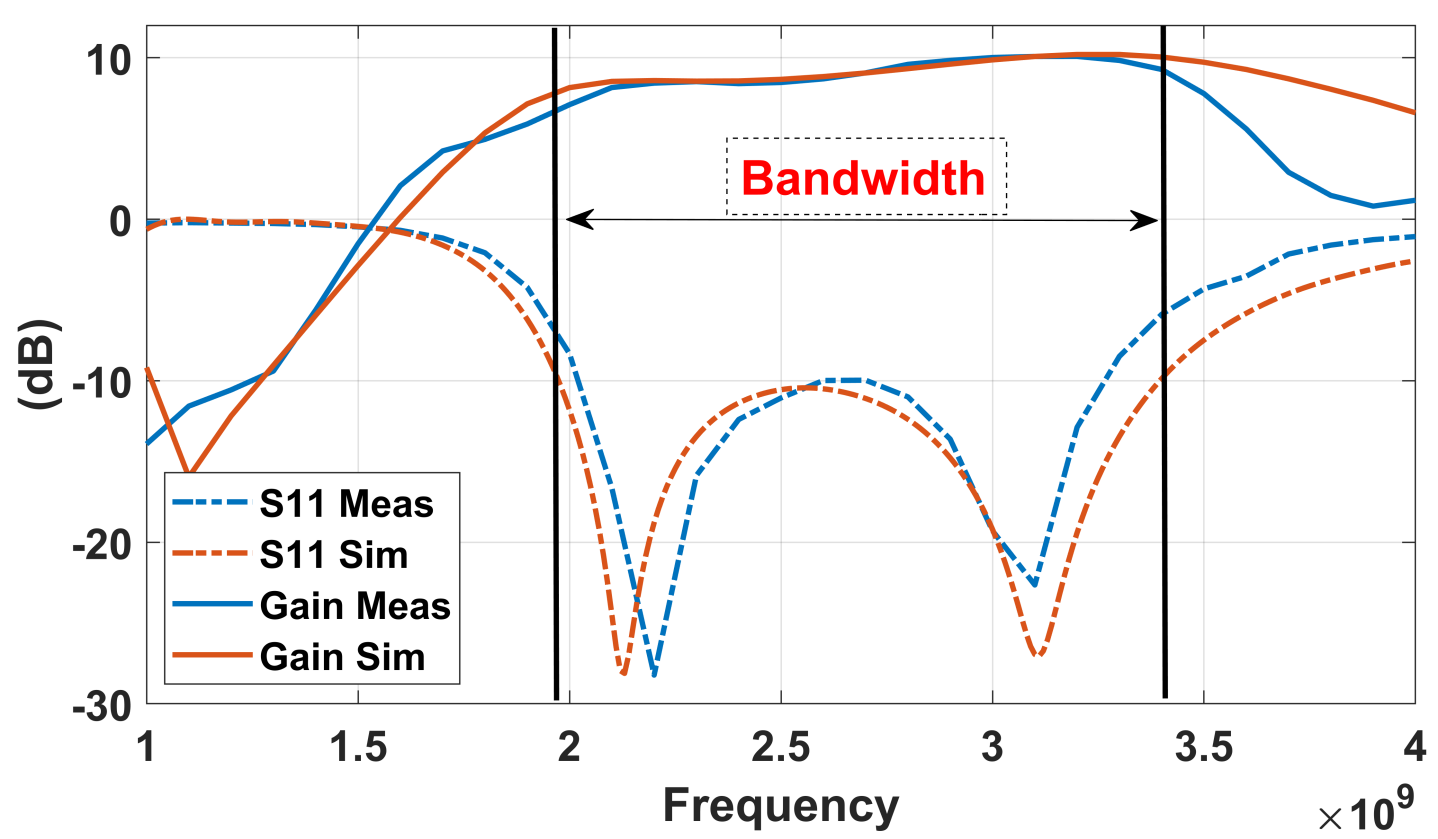
The $S_{11}$ and realized gain simulated and measured results of the prototype antenna.

After performing a comprehensive parametric study of the proposed proximity-coupled, cavity-less ME-dipole antenna, a new set of dimensions that provides a wider impedance bandwidth and more stable high gain than the initial model was obtained. A prototype was fabricated based on these dimensions, as shown in Figure 14. The $S_{11}$, gain, and radiation patterns of the prototype were then experimentally evaluated. As illustrated in Figure 15, the measured results of the prototype antenna agree reasonably with the simulation results. The measured impedance bandwidth is approximately 45.3% (from 2.05 to 3.25 GHz). The center frequency shifts by about 1.3%, and the impedance is narrower compared to the simulated results. The reduction in impedance bandwidth may be caused by height discrepancies in the manually fabricated prototype. Regarding the measured realized gain, the gain gradually increases from 8.0 dBi to 10.1 dBi, i.e., ±1.05 dBi within the passband, which is very close to the 8.0 dBi-to-10 dBi (±1.1 dBi) gain obtained in the simulation. A ground reflector, sized at approximately 0.83$\lambda_{0}^{2}$, was selected for the measurement.

**Figure 16:**
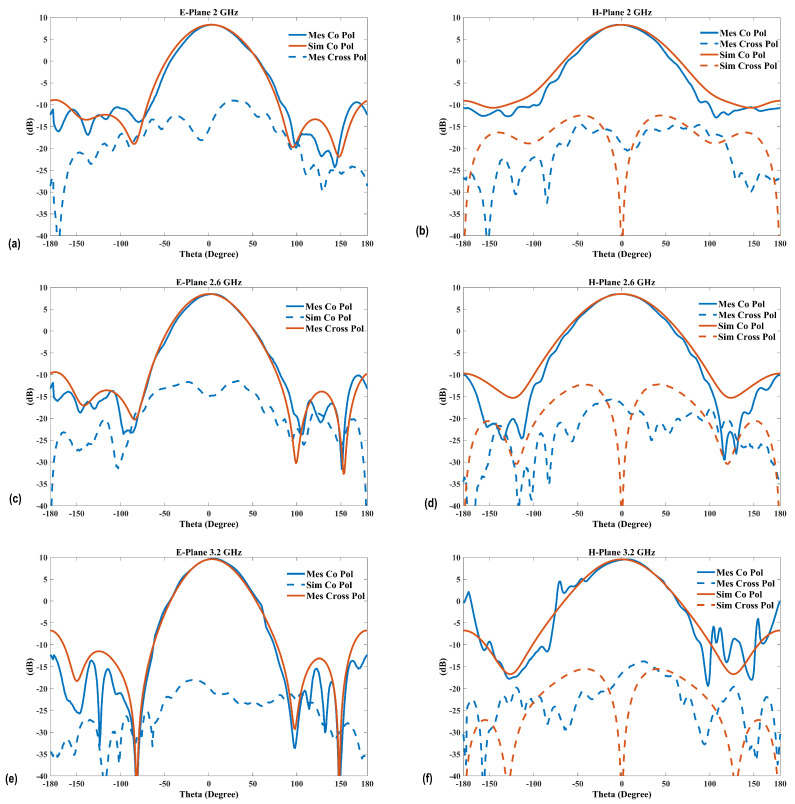
Measured and simulated radiation patterns of the optimized antenna: (**a**) E-plane at 2 GHz, (**b**) H-plane at 2 GHz, (**c**) E-plane at 2.6 GHz, (**d**) H-plane at 2.6 GHz, (**e**) E-plane at 3.2 GHz, and (**f**) H-planes at 3.2 GHz.

**Figure 17:**
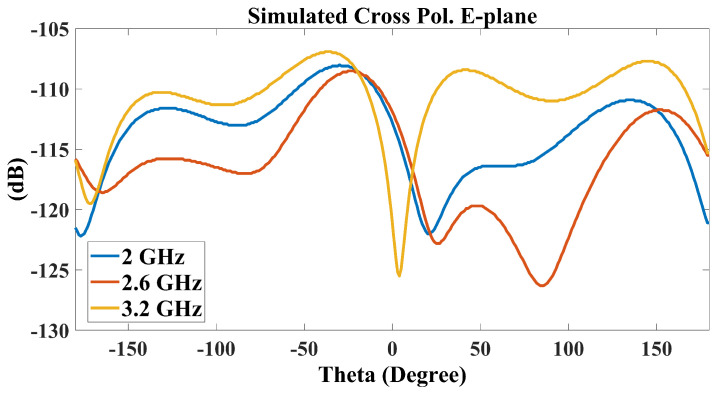
Simulated cross-polarization in the E-plane at 2 GHz, 2.6 GHz, and 3.2 GHz across the impedance bandwidth.

The simulated and measured radiation patterns covering the operational bandwidth at 2 GHz, 2.6 GHz, and 3.2 GHz are shown in Figure 16. It is observed that good agreement between the measurements and simulations is achieved. The unidirectional, stable radiation pattern with a low back-lobe of less than −20 dB, which is similar to conventional ME-dipole antennas, shows a reasonable cross-polarization level in the E-plane of about −20 dB. Moreover, since the simulation assumes a perfectly symmetrical structure and does not account for any fabrication errors, especially with the small 1 mm gap between the feed line and the radiation patches, the simulated cross-polarization level in the E-plane is below −100 dB. However, the measured cross-polarization is about −18 dB. Due to the ME-dipole operating concept, where the electric and M-dipole are oriented orthogonally, there is no effect on the antenna performance. Additionally, for reference, Figure 17 presents the simulated cross-polarization in the E-plane at different frequencies.

The radiation pattern summary is shown in Table 3. The front-to-back ratio is roughly 20 dB across the operational bandwidth. The 3 dB beamwidth differences across the operational bandwidth ($\Delta_{1}$) are 5 and 7.8 degrees in the H-plane and E-plane, respectively. The 3 dB beamwidth differences between both planes ($\Delta_{2}$) are 12.6, 13.2, and 22.75 degrees.

The proposed work was evaluated by comparing it with various ME-dipoles reported in the literature, as shown in Table 4. The proposed suspended-line coupling-fed ME-dipole achieves better-balanced antenna performance in terms of impedance bandwidth, stable high gain, and thickness. The proposed antenna exhibits a larger in-band gain variation, which is mainly caused by the lower gain contributed by the E-dipole, as shown in Figure 15. However, it retains a simpler design that eliminates the cavity walls for exciting the M-dipole. This simplification potentially reduces manufacturing costs.

## 6. Conclusions

A new design for a low-profile, proximity-coupled, cavity-less magneto-electric dipole antenna has been presented. This innovative design utilizes a coaxial cable and a suspended horizontal transmission line for feeding, effectively exciting the magneto-electric dipole without the need for quarter-wave cavity walls, which are typically used to excite the M-dipole. By eliminating the cavity walls, the proposed antenna simplifies the overall structure while maintaining very good performance characteristics. The antenna achieves a measured wide impedance bandwidth of 43.5%, which significantly enhances its operational flexibility, allowing it to cover a broad range of frequencies. Furthermore, the antenna delivers a maximum gain of 10.1 dBi, with an in-band gain variation of just 1.05 dBi, indicating stable performance across its operational bandwidth. This feeding approach not only optimizes the antenna’s performance but also enables a reduction in the size of the ground reflector to less than $1\lambda_{0}^{2}$, a size that is typically much larger in conventional designs. Despite the smaller ground reflector, the antenna maintains both a wide impedance bandwidth and high, stable gain across its entire operational bandwidth, highlighting the effectiveness and efficiency of the proposed design. This combination of reduced size and high performance makes the antenna suitable for a variety of applications where space and performance are critical factors.

## Figures and Tables

**Table 1:** Initial dimensions of the proposed ME-dipole antenna.

Parameter	Value (mm)	Parameter	Value (mm)
$L_{g}$	120	*H*	20
*L*	30	$H_{\mathrm{feed}}$	18
*W*	60	$L_{\mathrm{arm}}$	22
Gap	5	$H_{\mathrm{arm}}$	1

**Table 2:** Optimized dimensions of the proposed ME-dipole antenna.

Parameter	Value (mm)	Parameter	Value (mm)
$L_{g}$	120	*H*	20
*L*	29	$H_{\mathrm{feed}}$	18
*W*	42	$L_{\mathrm{arm}}$	22
Gap	3	$H_{\mathrm{arm}}$	1

**Table 3:** The radiation pattern summary, 3 dB beamwidth, and front-to-back ratio.

Frequency (GHz)	H Plane HPBW (°)	E Plane HPBW (°)	$\Delta_{2}$	Front-to-Back Ratio
2.0	59.7	72.3	12.6	18.7
2.6	56.0	69.2	13.2	18.9
3.2	54.2	64.3	10.1	22.5
$\Delta_{1}$	5.0	7.8	-	-

**Table 4:** Comparison of ME-dipole antenna designs.

Reference	$L_{g}$	Impedance Bandwidth (%)	Realized Gain (dBi)	In-Band Variation (dBi)	Height	Structure	Polarization
[1]	120 mm (1$\lambda_{0}$)	43.8	8.0	±0.4	0.25$\lambda_{0}$	Cavity walls	LP
[2]	180 mm (1.44$\lambda_{0}$)	86.9	7.2	±1.2	0.25$\lambda_{0}$	Cavity walls	LP
[3]	150 mm (1.28$\lambda_{0}$)	67.0	9.5	±2.5	0.25$\lambda_{0}$	Cavity walls	DP
[5]	120 mm (0.9$\lambda_{0}$)	72, 21	7.8	±1.3	0.25$\lambda_{0}$	Cavity walls	DP
[6]	150 mm (1.28$\lambda_{0}$)	34, 49.5	7.5	±0.5	0.25$\lambda_{0}$	Cavity walls	DP
[7]	112 mm (0.96$\lambda_{0}$)	28.2	9.2	±0.55	0.17$\lambda_{0}$	Cavity walls	LP
[8]	130 mm (1.01$\lambda_{0}$)	41.0	9.7	±0.45	0.16$\lambda_{0}$	Cavity-less	LP
[13]	112 mm (0.96$\lambda_{0}$)	54.8	8.6	±0.45	0.17$\lambda_{0}$	Cavity walls	LP
[14]	130 mm (1.04$\lambda_{0}$)	45.6	8.1	±0.4	0.17$\lambda_{0}$	Cavity walls	LP
[15]	155mm (1.75$\lambda_{0}$)	29.2	11.8	±1.7	0.6$\lambda_{0}$	Cavity walls	LP
[16]	230 mm (1.66$\lambda_{0}$)	89.3	9.8	±1.9	0.46$\lambda_{0}$	Cavity walls	LP
[17]	46 mm (0.58$\lambda_{0}$)	40	7.2	±0.5	0.1$\lambda_{0}$	Dielectric	DP
[18]	200 mm (1.83$\lambda_{0}$)	31.35	13.2	±1.5	0.57$\lambda_{0}$	Cavity walls	DP
[19]	110 mm (0.99$\lambda_{0}$)	43.6	9.3	±1	0.18$\lambda_{0}$	Cavity walls	CP
This work	100 mm (0.83$\lambda_{0}$)	45.3	9.0	±1.01	0.17$\lambda_{0}$	Cavity-less	LP

## Data Availability

Please contact Khalid Almegbel at khalid.almegbel.18@ucl.ac.uk.
